# A Bayesian model for identifying cancer subtypes from paired methylation profiles

**DOI:** 10.1093/bib/bbac568

**Published:** 2022-12-28

**Authors:** Yetian Fan, April S Chan, Jun Zhu, Suet Yi Leung, Xiaodan Fan

**Affiliations:** School of Mathematics and Statistics, Liaoning University, Shenyang, 110036, China; Department of Statistics, The Chinese University of Hong Kong, Sha Tin, New Territories, Hong Kong SAR, China; Department of Pathology, School of Clinical Medicine, The University of Hong Kong, Pokfulam, Hong Kong SAR, China; Sema4, Stamford, CT, 06902, USA; Icahn School of Medicine at Mount Sinai, New York, NY, USA; Department of Pathology, School of Clinical Medicine, The University of Hong Kong, Pokfulam, Hong Kong SAR, China; Department of Statistics, The Chinese University of Hong Kong, Sha Tin, New Territories, Hong Kong SAR, China

**Keywords:** cancer subtyping, Bayesian method, paired methylation profiles, clustering

## Abstract

Aberrant DNA methylation is the most common molecular lesion that is crucial for the occurrence and development of cancer, but has thus far been underappreciated as a clinical tool for cancer classification, diagnosis or as a guide for therapeutic decisions. Partly, this has been due to a lack of proven algorithms that can use methylation data to stratify patients into clinically relevant risk groups and subtypes that are of prognostic importance. Here, we proposed a novel Bayesian model to capture the methylation signatures of different subtypes from paired normal and tumor methylation array data. Application of our model to synthetic and empirical data showed high clustering accuracy, and was able to identify the possible epigenetic cause of a cancer subtype.

## Introduction

Cancers are highly complex and heterogeneous diseases. According to certain characteristics, including different histologies, molecular profiles and specific mutations, cancers can be grouped into different subtypes, which play a crucial role in providing proper treatment and prognosis [1–3]. For example, based on visual characteristics of tumor tissues under microscopes, a cancer can be divided into several clinically meaningful subtypes, under a procedure called a histological evaluation [4, 5]. For another instance, it is a fundamental and validated procedure to analyze gene expression profiles to identify cancer subtypes, which could be utilized as predictive markers to design personalized treatments [6–8]. Since genetic and epigenetic alternations are commonly known causal factors of cancers [9–12], grouping cancers according to different DNA methylation aberration patterns could promote the understanding of the critical role of epigenetic mechanisms in cellular processes and improve the effectiveness of cancer detection, diagnosis and prognosis [13–15].

Specifically, differential methylation analysis has shown that alterations in DNA methylation are closely associated with the occurrence and development of tumors [16–19]. Hypermethylation can result in gene silencing by repressing transcription of the promoter regions of tumor suppressor genes [20], whereas global hypomethylation can increase genomic and chromosomal instability [21, 22], both leading to cancer development. As some CpG islands are only methylated in specific tumor types, analysis of cancer type-specific differentially methylated regions revealed stochastic methylation variation that can be identified as signatures to distinguish different types of cancers [23]. Moreover, analysis of the distinct methylation patterns may distinguish different histological subtypes of lung cancers [24]. However, it is difficult to predict the clinical progression by current prognostic factors and treatments. But DNA methylation profiles can be used to identify tumor subtypes and provide a better understanding of individual tumor biology, which will help clinicians pick personalize patient treatment strategies [25]. All these studies suggest that the subtypes of cancers can be identified by the unique DNA methylation signature.

Recently, particularly promoted by high-throughput measurement technologies, a variety of computing methods have been applied to cluster cancers to subtypes. We will review some of the most related works. Auwera *et al*. [25] developed a statistical method to investigate some specific DNA methylation patterns, which was applied to distinguish subtypes of breast cancers. Rhee *et al*. [26] introduced an integrated approach combining DNA methylation and gene expression data to analyze breast cancers, where the results showed that methylation status may contribute to the inference of cancer subtypes. By analyzing the cancer cell-intrinsic signals from bulk tumor transcriptomic data, the computational deconvolution method DeClust stratifies patients into subtypes and provides the tumor-type-specific stromal profiles [27]. Zhang et al. [28] proposed InfiniumClust by applying mixture of Gaussian distributions to cluster cancer subtypes with consideration of tumor purity. Siegmund *et al*. [29] introduced a Bernoulli-lognormal mixture model to cluster the cancers based on DNA methylation data obtained by the quantitative MethyLight technology. Holm *et al*. [13] used K-means and hierarchical clustering to cluster cancers, and their results demonstrate that there are three groups of breast cancers with specific methylation profiles. Nonnegative matrix factorization method was applied to decompose DNA methylation profiles to categorize five consensus clusters, which were correlated with specific genetic abnormalities [30]. Furthermore, some methods integrated different types of data for clustering. For example, based on DNA methylation, gene expression, copy number, mutational and clinical data from pancreatic patients, Mishra and Guda [31] investigated the correlation between DNA methylation and differential gene expression profiles. For a more comprehensive review of the molecular subtyping methods, one can refer to Zhao *et al*. [32].

Different from subtyping methods which rely on predefined genes or regions, *de novo* subtyping methods automatically identify biomarkers for patient clustering in a data-driven fashion. Thus *de novo* methods are more useful in the scenario where the molecular mechanism is less clear, such as the effect of differential methylation. Some *de novo* subtyping methods are supervised learning method, which need a training dataset where the patients are well labeled to groups. Such supervised subtyping methods cannot help on complex diseases without enough labeling. A major problem of the existing *de novo* unsupervised subtyping methods is that the subtypes they infer may not be related to any disease mechanism, but purely correspond to different values of confounding variables such as age or race. To get rid of such confounding variables, more and more biomedical studies collected paired samples from tumor tissue and adjacent normal tissue, with the hope that the comparison between the paired samples can reveal how normal cells changed to cancer cells. A principled statistical method is needed to explicitly model such changes in order to efficiently detect the typical cancerization paths, where each path corresponds to a cancer subtype. In other words, traditional subtyping methods produce cancer status clusters, but we are interested in detecting aberrant-changes clusters.

In this paper, we propose a Bayesian mixture model for Subtyping (BaySub), which can provide the aberrant DNA methylation patterns of the cancer subtypes. Next, we describe our novel algorithm and demonstrate its performance in both synthetic and empirical data. The Bayesian estimation procedure for this model is provided in the Supplementary Information.

## Proposed method

### Data

We analyze paired DNA methylation array data from cancer patients. The original DNA methylation $\beta $-value is the ratio of the methylated probe intensity to the overall intensity, which ranges from 0 to 1. To better fit the Gaussian assumption of statistical modeling, M-value is more widely used to analyze differential methylation as it is a real number and approximately homoscedastic [33], which is defined as $M = log_2 (\frac{\beta }{1-\beta })$.

### Model

**Figure 1:**
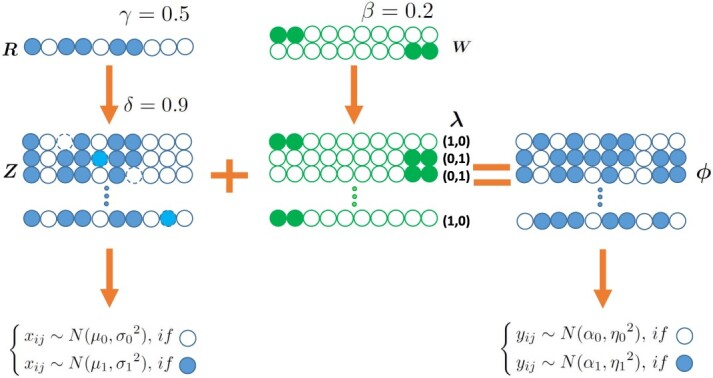
A flowchart to illustrate the BaySub algorithm. The methylation status of the reference normal cell is represented by a binary vector $\boldsymbol{R}$, and the rate of methylation is set to be $\gamma $. Based on this reference methylation status of normal tissue, a binary underlying methylation profile $\boldsymbol{Z}$ could be generated with mutation probability $1-\delta $. Suppose there are $s$ different paths $\boldsymbol{W}$ changing the methylation status from normal tissues to become tumor tissues, and the mutation rates is fixed at $\beta $. After obtaining the membership of the cancer subtypes $\boldsymbol{\lambda }$, the tumor methylation profile can be generated from normal methylation profile $\boldsymbol{Z}$, the membership $\boldsymbol{\lambda }$ and the mutation paths $\boldsymbol{W}$. Therefore, the M-values of normal tissues follow normal distribution according to its methylation profile $\boldsymbol{Z}$, and corresponding M-values of tumor tissues follow normal distribution according to its methylation profile $\boldsymbol{\phi }$.

The BaySub algorithm is illustrated in Figure 1. Suppose we have $n$ pairs of M-value vectors for matched tumor and normal samples, denoted by $D = \{x_i,y_i\}_{i=1}^n$, where $x_i$ and $y_i$ are both M-value vectors for $m$ CpG sites. We assume the $n$ normal samples are evolved from a reference normal cell with only non-disease-causing changes at a small percent of CpG sites. As a CpG site is either methylated or unmethylated, a binary variable $r_j$ following Bernoulli distribution is used to model the methylation status of the reference normal cell at the $j$-th CpG site:

$$ \begin{align*} r_j \sim i.i.d. \ Bernoulli(\gamma), \\ for \ j = 1,2,\dots,m, \end{align*} $$

which means the variable $r_j$ takes the value 1 with methylation probability $\gamma $.

For every normal tissue, we assume its binary underlying methylation profile $\{z_{ij}\}$ is generated from the reference normal cell profile $r_j$ with random turning over probability $1-\delta $:

$$ \begin{align*} z_{ij} = r_j \oplus Bernoulli(1-\delta), \\ for \ i = 1,2,\dots,n, \ j = 1,2,\dots,m, \end{align*} $$

where ‘$\oplus $’ stands for the exclusive-or operator.

For a normal tissue to become a tumor tissue, we assume that there are $s$ different paths from the corresponding normal tissue, each originated from a binary modification profile $\{w_{kj}\}$ and leading to a different subtype of the cancer of interest. More specifically, we assume the probability of turning over an original status in $z_{ij}$ is $\beta $, i.e.

$$ \begin{align*} w_{kj} \sim i.i.d. \ Bernoulli(\beta), \\ for \ k = 1,2,\dots,s, \ j = 1,2,\dots,m. \end{align*} $$

We assume each tumor sample contains one and only one subtype. Use a binary vector $\boldsymbol{\lambda _{i}}=(\lambda _{i1},\ldots ,\lambda _{is})$ to denote the signature membership of the $i$-th tumor, where $\lambda _{ik}=$ 0 or 1, $\sum _{k=1}^s \lambda _{ik} =1$. More specifically, we assume the signature follows multinomial distribution:

$$ \begin{align*} (\lambda_{i1},\ldots,\lambda_{is}) \sim Multinomial(1;p_1,p_2,\cdots,p_s), i =1,2,\dots,n. \end{align*} $$

The binary tumor methylation profile is assumed to be generated from the corresponding normal tissue profile with the perturbation at a subset of sites specified by the corresponding subtype signature:

$$ \begin{align*} \phi_{ij} = z_{ij} \oplus \sum_{k=1}^S \lambda_{ik} w_{kj}, &\\ for \ i = 1,2,\dots,n, \ j = 1,2,\dots,m, & \ k = 1,2,\dots,s. \end{align*} $$

Due to the methylation measurement error or the cell heterogeneity of a tissue, we assume the observation M-value follows a normal distribution centered around a typical value of the corresponding methylation status.

For the $i$-th normal tissue at the $j$-th CpG site, the measured M-value $x_{ij}$ is assumed to following $N(\mu _0, \sigma _0)$ if the corresponding $z_{ij}$ says it is unmethylated (i.e. $z_{ij}=0$), otherwise following $N(\mu _1, \sigma _1)$:

$$ \begin{align*} x_{ij} \sim N(\mu_0, {\sigma_0}^2)^{1-z_{ij}} \cdot N(\mu_1, {\sigma_1}^2)^{z_{ij}}, \\ for \ i = 1,2,\dots,n, \quad j = 1,2,\dots,m. \end{align*} $$

Similarly, we assume for the $i$-th tumor tissue at the $j$-th CpG site, the measured M-value $y_{ij}$:

$$ \begin{align*} y_{ij} \sim N(\alpha_0, {\eta_0}^2)^{1-\phi_{ij}} \cdot N(\alpha_1, {\eta_1}^2)^{\phi_{ij}}, \\ for \ i = 1,2,\dots,n, \quad j = 1,2,\dots,m. \end{align*} $$

In the above model, each row of the modification matrix $\boldsymbol{W}$ provides a unique path from a normal cell to a cancer cell, thus corresponding to one cancer subtype. If a cell in a row of $\boldsymbol{W}$ takes value 1, it means the methylation change of the corresponding CpG site is associated with the cancerization of that subtype. For individual patient, the corresponding $\boldsymbol{\lambda _i}$ specifies its subtype membership, which will be estimated from its posterior distribution using a maximum a-posteriori (MAP) approach.

For Bayesian inference, we introduced uniform priors and truncated uniform priors to ensure proper posterior purely determined by the data. Markov chain Monte Carlo (MCMC) algorithms are utilized to iteratively update all the parameters of our method until convergence. Detail distributions are given in the Supplementary Materials.

## Results

### Synthetic data experiments

In this section, we evaluate our method BaySub on the synthetic datasets. To check the effect of different number of true subtypes, we perform three experiments following our model based on the setting in Table 1. For each experiment, we repeat for 20 times independently, i.e. generating 20 independent and identically distributed datasets and estimating separately, to evaluate both the mean performance for this experiment and the performance variation. For the Bayesian inference for each dataset, we run 10 independent chains of our MCMC algorithm. The number of iterations for each run of the MCMC algorithm is set to 200, which turns out to be enough for convergence and posterior sample collection.

**Table 1:** Simulation settings

Parameters	Experiment 1	Experiment 2	Experiment 3
# Instance	100	100	100
# Subtypes	2	3	4
# CpG Sites	8000	8000	8000

**Figure 2:**
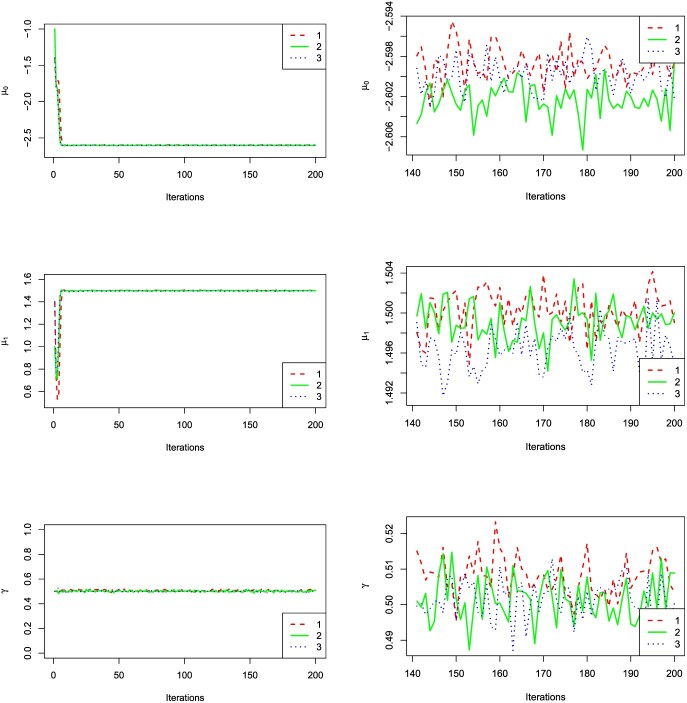
The traceplots of key variables for simulation datasets. The whole MCMC samples are shown in the left figures, and the burn-in periods are illustrated in the right figures.

For convergence diagnosis, the key variables $\gamma $, $\mu _0$ and $\mu _1$ are selected to show the convergence of our method. One of 20 trials is randomly selected from each experiment. Figure 2 describes the changes of variable with the increasing of iterations. The results reveal that our method converges rapidly and stably. Besides, the Gelman–Rubin convergence diagnostic ($\hat{R}$) is calculated to evaluate the convergence of MCMC. The values of variables selected from the last 60 iterations are divided into two sequences of length 30. It turns out that all the $\hat{R}$ values are smaller than 1.1, which indicates adequate convergence. Thus, both Figure 2 and Table 2 show rapid convergence of our algorithm.

**Table 2:** The $\hat{R}$ values of key parameters for the three simulation experiments. A $\hat{R}$ value closer to 1 indicates good convergence of the MCMC algorithm

Parameter/experiment	1	2	3
$\mu _0$	1.038	1.024	1.044
$\mu _1$	1.002	0.993	1.067
$\gamma $	1.090	0.994	0.991

We adopt two popular clustering performance measures, adjusted Rand index (ARI) and normalize mutual information (NMI), to evaluate the clustering performance of our method [34, 35]. Moreover, we use two measures to quantify the inference accuracy of differential methylation. Based on the true value of aberrant methylation signatures $\tilde{\boldsymbol{W}}$, we calculate the inference accuracy of all elements in the variable $\boldsymbol{W}$ (denoted by AE), defined as

$$ \begin{align*} \frac{1}{s\times m} \sum_{k=1}^s \sum_{j=1}^m I(w_{kj} = \tilde{w}_{kj}). \end{align*} $$

Alternatively, we measure the site-wise inference accuracy by the percent of CpG sites whose status are totally correctly inferred (denoted by AS), i.e.

$$ \begin{align*} \frac{1}{m} \sum_{j=1}^m \prod_{k=1}^s I(w_{kj} = \tilde{w}_{kj}). \end{align*} $$

Based on the above measurement, three synthetic datasets are utilized to evaluate the performance of BaySub method. As each experiment is repeated for 20 times independently, the mean performance and standard deviation are summarized in Table 3, which illustrates that our method obtains a high accuracy for both detecting cancer subtypes and identifying the methylation patterns.

**Table 3:** Performance of BaySub on simulation datasets. The numbers in the brackets are the standard deviation of corresponding performance measures based on 20 independent trials

Experiment	Subtypes	ARI	NMI	AE(%)	AS(%)
1	2	1 (0.00)	1 (0.00)	99.91 (0.18)	99.90 (0.18)
2	3	1 (0.00)	1 (0.00)	99.99 (0.01)	99.98 (0.03)
3	4	1 (0.00)	1 (0.00)	99.98 (0.02)	99.93 (0.07)

**Figure 3:**
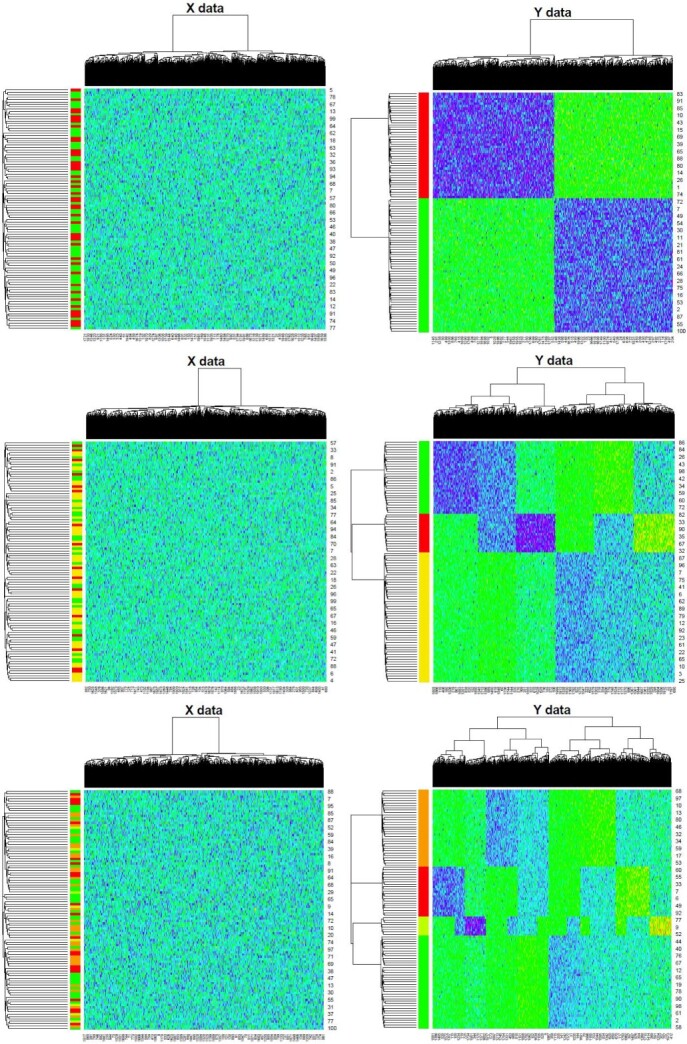
Heatmap of methylation signatures captured by BaySub algorithm on simulation datasets.

For visualization, we plot the heatmap in Figure 3 to demonstrate the selected CpG sites, which can capture the specific methylation signatures associated with different cancer subtypes. For clarity, we only plot those differentially methylated CpG sites on which the value of corresponding elements in $\boldsymbol{W}$ should be 1 (i.e. differentially methylated between normal and tumor tissues). The colors in the left bar represent the true subtypes. From the figure, it is easy to see some block structures in heatmap, which indicates that these data include several methylation patterns.

Since each experiment is repeated for 20 times based on 20 independent datasets synthesized from a same parameter setting, after burn in, the estimates of all the variables in every trial are calculated by the mean of the last 30 iterations. Then the accuracy of variables can be checked by the mean and the responding standard deviation, which are the point estimates from the 20 independent trials. Table 4 presents the variables estimated by our method. The results show that our estimating method preforms well on these simulation datasets.

**Table 4:** Results of parameters estimation on simulation datasets. The numbers in the brackets are the standard deviation of corresponding estimates based on 20 independent trials

Parameters	True Value	Experiment 1	Experiment 2	Experiment 3
$\mu _0$	–2.6	–2.600 (0.002)	–2.600 (0.002)	–2.600 (0.002)
$\mu _1$	1.5	1.494 (0.012)	1.500 (0.002)	1.499 (0.002)
$\sigma _0$	1.3	1.299 (0.003)	1.300 (0.003)	1.301 (0.003)
$\sigma _1$	1.4	1.422 (0.039)	1.401 (0.003)	1.400 (0.003)
$\alpha _0$	–2.9	–2.903 (0.01)	–2.902 (0.007)	–2.897 (0.008)
$\alpha _1$	1.1	1.101 (0.012)	1.103 (0.007)	1.101 (0.009)
$\eta _0$	1.1	1.099 (0.002)	1.100 (0.003)	1.102 (0.003)
$\eta _1$	1.7	1.703 (0.006)	1.699 (0.005)	1.701 (0.003)
$\gamma $	0.5	0.501 (0.006)	0.500 (0.006)	0.501 (0.006)
$\delta $	0.99	0.990 (0.000)	0.990 (0.000)	0.990 (0.000)
$\beta $	0.2	0.202 (0.003)	0.201 (0.003)	0.200 (0.003)

**Figure 4:**
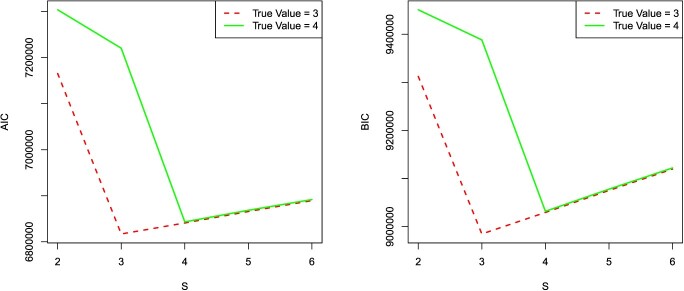
The plots of AIC (left) and BIC (right) for different numbers of subtypes in Experiment 2 (dashed line) and Experiment 3 (solid line). The horizontal axis represents the assumed value of the number of subtypes $s$, and the vertical axis represents the corresponding values of AIC or BIC. The true value of $s$ for red dashed line is 3, and that for green solid line is 4.

In our method, the number of subtypes $s$ should be given in advance, nevertheless it might be difficult to obtain the accurate value for real datasets. Here, Akaike information criterion (AIC) and Bayesian information criterion (BIC) are utilized to seek an appropriate model by compromising the goodness-of-fit and model complexity. We calculate the AIC and BIC values of the model with $s$ values ranging from 2 to 6 on Experiment 2 and Experiment 3 datasets. The results are demonstrated in Figure 4, which shows that the minimum values corresponds with the true number of subtypes for both AIC and BIC. Hence, $s$ can be readily learnt from the data.

One may still worry a wrongly specified number of subtypes may lead to meaningless results. To address this concern, we also test our method with a wrong number of cancer subtypes. We randomly select two datasets from Experiment 2 and Experiment 3 separately, and set the value of $s$ to be the true value plus or minus 1. The experiments results are shown in Table 5. For example, when the $s$ value is set to be 2 but the true value is 3, the first and third true subtypes are merged into one inferred group, which is marked by $\tilde{1}$. For another instance, when the $s$ value is set to be 4 but the true value is 3, our algorithm may lead to two prediction results. In the first result, the inferred subtype $\tilde{3}$ is actually an empty class (thus purely dominated by the prior without support from the data), while the other three inferred subtypes correspond to the three true subtypes perfectly. In the second result, the third true subtype is divided into the two inferred subtype $\tilde{3}$ and $\tilde{4}$. All the results demonstrate that our method BaySub could deal with a slightly wrong number of cancer subtypes reasonably.

**Table 5:** Performance of BaySub with a wrongly specified number of cancer subtypes. The true number of subtypes is shown in the first column, and the used values of $s$ by our method for inference are displayed in the second column. The relations between true membership and predicted membership are provided in the last column. The numbers on the left and right of the symbol ‘$\sim $’ represent the true and inferred membership, respectively. The percentage in the bracket indicates the proportion of the predicted membership involved in the corresponding matching. For example, ‘$3 \sim \tilde{1}$(65.2%)’ means 65.2% of the patients in the inferred subtype 1 are actually from the true subtype 3

Subtypes	Used $s$	Matching results
3	2	$1 \sim \tilde{1}$ (34.8%), $2 \sim \tilde{2}$(100%), $3 \sim \tilde{1}$(65.2%)
3	4	$1 \sim \tilde{1}$ (100%), $2 \sim \tilde{2}$(100%), $3 \sim \tilde{4}$(100%)
		or $1 \sim \tilde{1}$(100%), $2 \sim \tilde{2}$(100%), $3 \sim \tilde{3}$(100%), $3 \sim \tilde{4}$(100%)
4	3	$1 \sim \tilde{1}$ (39.6%), $2 \sim \tilde{1}$(60.4%), $3 \sim \tilde{2}$(100%), $4 \sim \tilde{3}$(100%)
4	5	$1 \sim \tilde{1}$ (100%), $2 \sim \tilde{5}$(100%), $3 \sim \tilde{3}$(100%), $4 \sim \tilde{4}$(100%)
		or $1 \sim \tilde{1}$(100%), $2 \sim \tilde{2}$(100%), $3 \sim \tilde{3}$(100%), $4 \sim \tilde{4}$(100%), $1 \sim \tilde{5}$(100%)

As tumor heterogeneity affects the assignment of subtypes in the clinic, it is commonly concerned during data analysis that tumor tissues contain multiple cancer subtypes or are contaminated by normal cells during dissection. If every tumor contains more than one subtype, it would be more challenging for our method BaySub to deal with such a mixture of different subtypes and normal data. To check the performance of our method on ‘contaminated’ datasets, we run 9 extra experiments with different numbers of true subtypes and different component proportions. Suppose there are normal tissues and $s$ subtypes of tumor tissues; each of them have $n$ pairs of methylation vectors for $m$ CpG sites, denoted by $\{x_{k,i},y_{k,i}\}$, where $x_{k,i} \in \mathbb{R}^m, y_{k,i} \in \mathbb{R}^m, i=1,2,\dots ,n,k=0,1,\dots ,s$, and $k=0$ represents normal tissues, for different tumor tissues, $k=1,2,\dots ,s$. Then a mixture dataset could be generated by integrating different subtypes and normal data by following steps: for the $i$-th paired mixture methylation vectors $\{x_i,y_i\}_{i=1}^n,x_{i} \in \mathbb{R}^m, y_{i} \in \mathbb{R}^m$, first, two subtypes of cancers are randomly selected from all the subtypes (denoted by subtype $A_i$ and $B_i$), which are randomly selected from the subtypes $\{1,2,\dots ,s\}$. Second, the $i$-th paired methylation vectors is a linear combination of tumor tissues from these two selected subtypes and normal tissues, whose proportion is represented by a vector $(P_A,P_B,P_N)$, i.e. $\{x_i,y_i\}= P_A \times \{x_{A_i,i},y_{A_i,i}\} + P_B\times \{x_{B_i,i},y_{B_i,i}\} + P_N \times \{x_{0,i},y_{0,i}\}$. The predicting target is set to be the major subtype, which is the largest proportion. Last, we repeat these two procedures to obtain the ‘contaminated’ datasets until the number of pairs equals 100. The results are shown in Table 6, which illustrate that our method could still achieve high accuracy without significant deterioration by these ‘contaminated’ data when one subtype gains an outright majority, while the predicting accuracy would decrease with the gradual disappearance of this majority advantage.

**Table 6:** Performance of BaySub on ‘contaminated’ datasets. The true number of subtypes is reported in the first column. The proportions of $(P_A,P_B,P_N)$ are shown in the second column. The biggest number is the proportion of major subtype, which is the predicting target. All the accuracies are displayed in the third to sixth columns

Subtypes	Proportions	ARI	NMI	AE(%)	AS(%)
2	(0.8,0.1,0.1)	1	1	99.56	99.54
2	(0.6,0.2,0.2)	1	1	100	100
2	(0.4,0.3,0.3)	0.2981	0.2914	91.73	83.65
3	(0.8,0.1,0.1)	1	1	100	99.99
3	(0.6,0.2,0.2)	1	1	99.73	99.20
3	(0.4,0.3,0.3)	0.2417	0.3622	83.81	51.44
4	(0.8,0.1,0.1)	1	1	99.98	99.91
4	(0.6,0.2,0.2)	1	1	99.73	98.93
4	(0.4,0.3,0.3)	0.2776	0.4213	84.32	41.86

In our model, we assume that the methylation value of every CpG site is independent and identically distributed (i.i.d.), and the distributions of methylation profiles for both tumor and normal tissues follow the Gaussian distribution. In this part, we will evaluate the performance of our method BaySub on the simulation datasets, which are generated from t distribution and the methylation status of the tumor and normal tissues are dependent. First, the vector $\boldsymbol{R}$, representing the methylation status of the reference normal cell, is separated into 100 regions randomly. For every CpG site, the methylation status takes the value 1 with a probability of 0.7 or 0.3, and the probabilities for two adjacent regions are different. Second, for every row of modification matrix $\boldsymbol{W}$, it is also separated into 100 regions randomly. The values within one region are the same. They take the value 1 with a probability of 0.2. Therefore, the elements in both vector $\boldsymbol{R}$ and matrix $\boldsymbol{W}$ are dependent, so the generated methylation profiles for tumor and normal tissues are dependent. The other parameters are the same as the values shown in Table 4. Although the BaySub algorithm is based on the assumption of i.i.d. and normal distribution, the experiment results illustrated in Table 7 verify that it could obtain high prediction accuracy and extract meaningful methylation patterns from the whole picture of cancer.

**Table 7:** Performance of BaySub on t distribution and correlated methylation levels

Experiment	# Instance	# Subtypes	# CpG Sites	ARI	NMI	AE(%)	AS(%)
1	100	2	8000	1	1	99.91	99.81
2	100	3	8000	1	1	98.47	95.46
3	100	4	8000	1	1	98.28	93.27

### Empirical data experiments

In this section, we evaluate the performance of our method BaySub on the real data of human cancer. We downloaded the methylation data from both the research on whole-genome sequencing in gastric cancer and TCGA database, which contain the methylation array data from both tumor and corresponding normal tissues [36]. All the DNA methylation profiles are measured by the same probe layout of 450K array, which is the most widely used platform for DNA methylation. Based on the research, gastric cancer is a heterogeneous disease, which can be separated into three different molecular subgroups: microsatellite instability (denoted by MSI), Epstein–Barr virus (denoted by EBV) and others. For TCGA database, based on the primary sites, these datasets are used to compose the second trial. Since these methylation profiles have some missing data, the common CpG sites shared by all datasets in the corresponding trial will be applied to infer the cancer subtypes. More details of every trial are shown in Table 8. The number of iterations is 200. The number of chains is set to 10.

**Table 8:** Specification of real datasets

Trial index	Primary site	Data Project	Subtypes	Pairs	# CpG sites
1	Gastric	Gastric Cancer	MSI / EBV / others	6+3+24	459 158
2	Esophagus	ESCA	Squamous Cell Neoplasms / Adenomas and Adenocarcinomas	3+12	390 481

For the evaluation of the predicting accuracy, the true molecular subgroup of human cancer can be utilized to evaluate the estimated variable $\boldsymbol{\lambda }$. To evaluate the achieved methylation signatures $\boldsymbol{W}$, we centralized all the CpG sites for every cancer subtype to generate a standard value $\tilde{\boldsymbol{W}}$. First, we calculate the mean M-value vectors of every cancer subtype on all $m$ CpG sites, denoted by $\{\overline{x_k},\overline{y_k}\}$, where $\overline{x_k} \in \mathbb{R}^m,\overline{y_k} \in \mathbb{R}^m,k=1,2,\dots ,s$. Then, based on the definition of M-value, 0 is selected as the segmentation criterion to divide all CpG sites for the every cancer subtype into hypermethylation and hypomethylation. For example, for the $j$-th CpG site of $k$ subtype, the average status of methylation for tumor is represented by $c_{k,j}^y = 1$, if $\overline{y_{k,j}}>0$, otherwise $c_{k,j}^y = 0$, and it is the same with the average methylation status for normal data $c_{k,j}^x$. Therefore, the average status of methylation on all CpG sites for every subtype can be represented by $\{c_{k,j}^x,c_{k,j}^y\}$, where $j=1,2,\dots ,m,k=1,2,\dots ,s$. Finally, we can calculate the target variable $\tilde{\boldsymbol{W}} = (\tilde{w}_{kj})_{s\times m}$, where $\tilde{w}_{kj}= c_{k,j}^x \oplus c_{k,j}^y$. Based on the obtained $\tilde{\boldsymbol{W}}$, we can calculate both the accuracy of elements and the accuracy of CpG sites in variable $W$. The comparison results are shown in Table 9, which illustrates the good performance of our method BaySub on both predicting accuracy and identifying methylation patterns of different cancer subtypes. The posterior estimate of key parameters are listed in Table 10, including posterior mean and standard deviation.

**Table 9:** Performance of BaySub on real datasets

Trial index	ARI	NMI	AE(%)	AS(%)
1	0.1922	0.1477	88.86	78.21
2	0.1791	0.2630	86.06	78.18

**Table 10:** Results of parameters estimation on real datasets. The numbers in the brackets are the standard deviation

Parameter/Trial	1	2
$\mu _0$	–3.2087 (0.0011)	–2.7211 (0.0010)
$\mu _1$	1.9599 (0.0004)	1.3372 (0.0008)
$\sigma _0$	6.4645 (0.0039)	1.4978 (0.0028)
$\sigma _1$	1.5536 (0.0006)	1.5689 (0.0014)
$\alpha _0$	–3.5837 (0.0026)	–2.9001 (0.0132)
$\alpha _1$	1.4812 (0.0019)	0.9904 (0.0000)
$\eta _0$	7.3066 (0.0046)	1.2662 (0.0124)
$\eta _1$	1.9583 (0.0011)	1.7434 (0.0061)
$\gamma $	0.5530 (0.0008)	0.5297 (0.0008)
$\delta $	0.9996 (0.0000)	0.9927 (0.0001)
$\beta $	0.0708 (0.0003)	0.0920 (0.0011)

**Figure 5:**
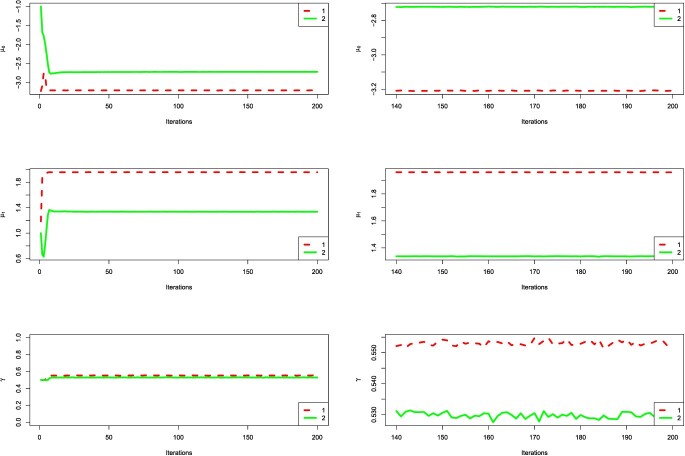
The traceplots of key variables for real datasets. The whole MCMC samples are shown in the left figures, and the burn-in periods are illustrated in the right figures.

Last, we also analyze the convergence of our method on the two real datasets. Both Figure 5 and Table 11 show that our method on real datasets converges rapidly.

**Table 11:** The $\hat{R}$ values of key parameter for real datasets

Parameter/Experiment	1	2
$\mu _0$	1.001	1.044
$\mu _1$	1.015	1.067
$\gamma $	1.021	0.991

**Table 12:** Performance comparison on ‘pure’ datasets

Subtypes	Modified Parameters	ARI						NMI					
		BaySub	K-means	PAM	CLARA	HC	NMF	BaySub	K-means	PAM	CLARA	HC	NMF
4	$\mu _0 = -30, \mu _1 = 30, \sigma _0 =30, \sigma _1=30, \beta =0.2$	1	0.8582	0.2858	0.4851	0.9224	0.0353	1	0.8848	0.4151	0.5811	0.8689	0.0854
4	$\alpha _0 = -30, \alpha _1 = 30, \eta _0 =30, \eta _1=30,\beta =0.2$	1	0.9080	1	1	1	0.9678	1	0.9228	1	1	1	0.9648
4	$\mu _0 = -30, \mu _1 = 30, \sigma _0 =30, \sigma _1=30,\beta =0.05$	1	0.7246	0.0686	0.0246	0.1822	0.0109	1	0.7009	0.1388	0.0448	0.1370	0.0524
4	$\alpha _0 = -30, \alpha _1 = 30, \eta _0 =30, \eta _1=30,\beta =0.05$	1	0.9284	1	1	1	0.8492	1	0.9360	1	1	1	0.8318
4	$\mu _0 = -30, \mu _1 = 30, \sigma _0 =30, \sigma _1=30,\beta =0.01$	1	0.0233	–0.0076	–0.0076	0.0048	0.0018	1	0.0575	0.0255	0.0255	0.0346	0.0385
4	$\alpha _0 = -30, \alpha _1 = 30, \eta _0 =30, \eta _1=30,\beta =0.01$	1	0.8559	1	1	1	0.6921	1	0.8802	1	1	1	0.7219

**Table 13:** Performance comparison on ‘contaminated’ datasets

Subtypes	Proportion	Modified parameters	ARI						NMI					
			BaySub	K-means	PAM	CLARA	HC	NMF	BaySub	K-means	PAM	CLARA	HC	NMF
4	(0.8,0.1,0.1)	$\beta =0.2, \delta =0.9$	1	0.8876	1	1	1	0.4496	1	0.9227	1	1	1	0.5309
4	(0.6,0.2,0.2)	$\beta =0.2, \delta =0.9$	1	0.8924	1	1	1	0.2867	1	0.9212	1	1	1	0.3945
4	(0.4,0.3,0.3)	$\beta =0.2, \delta =0.9$	0.7338	0.2951	0.3055	0.2531	0.2266	0.2050	0.7643	0.4058	0.4215	0.3948	0.3497	0.3006
4	(0.8,0.1,0.1)	$\beta =0.05, \delta =0.9$	0.9734	0.9439	0.9862	1	1	0.1805	0.9442	0.9584	0.9667	1	1	0.2584
4	(0.6,0.2,0.2)	$\beta =0.05, \delta =0.9$	1	0.9421	0.9409	0.9554	1	0.1674	1	0.9538	0.9209	0.9323	1	0.2405
4	(0.4,0.3,0.3)	$\beta =0.05, \delta =0.9$	0.6386	0.3067	0.2784	0.1865	0.1955	0.0934	0.6421	0.4074	0.2931	0.2329	0.2864	0.1474
4	(0.8,0.1,0.1)	$\beta =0.01, \delta =0.9$	0.8088	0.8247	0.1276	0.1276	0.2776	0.0265	0.7916	0.8483	0.1645	0.1645	0.3664	0.0661
4	(0.6,0.2,0.2)	$\beta =0.01, \delta =0.9$	0.7821	0.5975	0.0734	0.0718	0.2742	0.0166	0.7515	0.5956	0.1315	0.1048	0.3001	0.0563
4	(0.4,0.3,0.3)	$\beta =0.01, \delta =0.9$	0.0417	0.0668	0.0208	0.0118	0.0339	0.0063	0.2059	0.1159	0.0787	0.0548	0.0533	0.0437

### Performance comparison

In this section, we compare the performance of BaySub with other popular clustering methods: K-means [37], K-medoids (PAM, CLARA) [38], Hierarchical Clustering (denoted by HC) [39] and Non-negative Matrix Factorization (denoted by NMF) [40]. As the clustering results of algorithms K-means and NMF are unstable due to different initial values, the average accuracy of 100 trials is reported as the comparison performance. The values of $s$ for all methods are set to be 4. First, we compare the accuracy of these methods on ‘pure’ datasets (every tumor contains only one subtype). Most parameters are the same with the values of the simulation datasets, which are illustrated in Table 4. To improve the diversity of methylation statuses for tumor and normal tissues, the mean and the variance for normal tissue ($\mu $, $\sigma $) and tumor tissue ($\alpha $, $\eta $) are increased, respectively. The value of $\beta $ is decreased from 0.2 to 0.05 to reduce the difference between cancer subtypes. Based on Table 12, these factors have little influence on our method BaySub, while the performances of other methods decline dramatically. Moreover, we also compare the performance of BaySub with other clustering methods on ‘contaminated’ datasets. We change the purity of tumor by reducing the dominance of major subtype, and alter the probability $\beta $ turning over on the modification profile $w$. The comparison results in Table 13 display that the purity and the difference effect the performances of both BaySub and other clustering methods, but the predicting accuracy of BaySub method outperforms others. Last, we compare the performance of BaySub with other methods on empirical data, whose results are shown in Table 14. In conclusion, based on Tables 12–14, our method BaySub obtains better performance than some other clustering methods on both synthetic and empirical datasets.

**Table 14:** Performance comparison on empirical datasets

Trial Index	Subtypes	ARI						NMI					
		BaySub	K-means	PAM	CLARA	HC	NMF	BaySub	K-means	PAM	CLARA	HC	NMF
1	3	0.1922	0.0213	–0.0823	–0.0823	–0.1042	0.1193	0.1477	0.2009	0.0264	0.0264	0.0838	0.1415
2	2	0.1791	0.1652	–0.1320	–0.1320	–0.1320	0.0272	0.2630	0.0907	0.0644	0.0644	0.0644	0.0438

## Conclusions

In this paper, we propose a Bayesian mixture model named BaySub for predicting the cancer subtypes based on paired methylation array data. Our method can capture the methylation signatures for different cancer subtypes. We evaluate the performance of our method on both synthetic and empirical data experiments. In synthetic data experiments, our method achieve high predicting accuracy. Besides, the results of these experiments reveal that the proposed algorithm has good robustness and convergence. Moreover, our method can not only seek an appropriate number of subtypes by AIC and BIC values, but also can deal with the wrong specification of the number of subtypes. In real data experiments, the performance of our method is evaluated on two datasets, which are generated from both the gastric cancer project and TCGA database. The results illustrate the good performance of our method on real datasets, and the estimated parameters of the model converge rapidly and stably. Furthermore, in the section of performance comparison, BaySub algorithm provides better performance than some other clustering methods on both synthetic and empirical datasets. Note that BaySub can essentially deal with any disease where methylation changes play an important role, as long as each patient provides both a normal and a disease sample for methylation profiling.

There are several directions to improve our method. First of all, we assume the methylation values of every CpG site are independent and identically distributed (i.i.d.) to reduce the complexity of model. Although simulation experiments show that if the simulated datasets are generated from heavy tail distribution and correlated methylation levels, BaySub based on the i.i.d. assumption could still extract meaningful information from the whole picture of simulated datasets, methylation statuses of neighboring sites seem positively correlated in reality instead of independent. Thus, future studies on releasing this i.i.d. assumption may contribute to improving the performance of cancer subtyping. Secondly, the current version of BaySub is designed for the methylation array data, but the binary methylation profiles used in BaySub essentially fits the single-cell methylation data well. One may modify the Gaussian observation model to adapt BaySub for single-cell methylation data analyses. Moreover, BaySub is currently designed for paired methylation data from tumors and adjacent normal tissues. In real tumor studies, not all patients can provide paired samples. Thus, it is important to extend BaySub by integrating unpaired and paired samples.

**Key Points**

- We propose a new Bayesian method named BaySub for clustering cancer patients based on paired methylation array data, which can simultaneously identify the methylation changes associated with different cancer subtypes.
- Our method can not only seek an appropriate number of clusters through AIC and BIC, but also properly handle the wrong specification of the number of subtypes.
- We evaluated the performance of our method based on both synthetic and empirical data, which showed that our method could achieve high clustering accuracy.

## Author Biographies

**Yetian Fan** is a lecturer at School of Mathematics and Statistics, Liaoning University, and a postdoctoral fellow at Department of Statistics, The Chinese University of Hong Kong.

**April S Chan** is a research associate at Department of Pathology, School of Clinical Medicine, The University of Hong Kong.

**Jun Zhu** is a professor at Icahn School of Medicine at Mount Sinai and Sema4.

**Suet Yi Leung** is a professor at Department of Pathology, School of Clinical Medicine, The University of Hong Kong.

**Xiaodan Fan** is a professor at Department of Statistics, The Chinese University of Hong Kong.

## A Appendix

### A.1 Bayesian inference

In this section, we introduce the Bayesian approach to estimate the parameter for our proposed model. For Bayesian inference, we assign uniform priors to most parameters except for the $\mu _c$, $\sigma _c^2$, $\alpha _c$ and $\eta _c^2$. Truncated uniform priors are used for these four parameters, and the truncation for $\mu _c$ and $\alpha _c$ is defined by the boundaries (-50,50)which for $\sigma _c^2$ and $\eta _c^2$ is (0,100). With all the above assumptions and uninformative priors[41], we can get the conditional distributions of variables:

$$ \begin{align*} r_j |\gamma &\sim i.i.d. \ Bernoulli(\gamma) \\ w_{kj}|\beta &\sim i.i.d. \ Bernoulli(\beta) \\ (\lambda_{i1},\ldots,\lambda_{is})|\boldsymbol{P} &\sim Multinomial(1;p_1,p_2,\cdots,p_s) \\ z_{ij}|r_j,\delta &= r_j \oplus Bernoulli(1-\delta) \\ x_{ij}|z_{ij},\mu_0,\mu_1,\sigma_0,\sigma_1 &\sim i.i.d. \ N(\mu_0, {\sigma_0}^2)^{1-z_{ij}} \cdot N(\mu_1, {\sigma_1}^2)^{z_{ij}} \\ \phi_{ij}|z_{ij},\lambda_{ik},w_{kj} &= z_{ij} \oplus \sum_{k=1}^S \lambda_{ik} w_{kj} \\ y_{ij}|\phi_{ij},\alpha_0,\alpha_1,\eta_0,\eta_1 &\sim i.i.d. \ N(\alpha_0, {\eta_0}^2)^{1-\phi_{ij}} \cdot N(\alpha_1, {\eta_1}^2)^{\phi_{ij}} \\ \end{align*} $$

Therefore, the full posterior distribution $f(\boldsymbol{X},\boldsymbol{Y};\boldsymbol{\theta })$ is proportional to

$$ \begin{align*} &\prod_{i=1}^n \prod_{j=1}^m \prod_{c=0}^1 \left[\frac{1}{\sqrt{2 \pi \eta_c^2}} exp \left\{ - \frac{(y_{ij}-\alpha_c)^2}{2 \eta_c^2} \right\} \right] ^ {I(\phi_{ij} = c)} \\ &*\prod_{i=1}^n \prod_{j=1}^m \prod_{c=0}^1 \left[\frac{1}{\sqrt{2 \pi \sigma_c^2}} exp\left \{ - \frac{(x_{ij}-\mu_c)^2}{2 \sigma_c^2}\right \} \right] ^ {I(z_{ij} = c)} \\ &*\prod_{i=1}^n p_1^{I(\lambda_{i1}=1)} p_2^{I(\lambda_{i2}=1)} \cdots p_s^{I(\lambda_{is}=1)} \\ &*\prod_{i=1}^n \prod_{j=1}^m \delta^{I(z_{ij}=r_j)} (1 - \delta)^{I(z_{ij}=1 - r_j)} \\ &*\prod_{j=1}^m \gamma^{I(r_j=1)} (1 - \gamma)^{I(r_j=0)} \\ &*\prod_{k=1}^s \prod_{j=1}^m \beta^{I(w_{kj}=1)} (1 - \beta)^{I(w_{kj}=0)} \end{align*} $$

All the parameters of our method are iteratively updated from their corresponding posterior distribution by the Metropolis-within-Gibbs algorithms. The algorithm is summarized as follows:

1. Initialize all the parameters $\gamma ^{[1]}$,$\beta ^{[1]}$,$\delta ^{[1]}$,$\boldsymbol{R}^{[1]}$,$\boldsymbol{P}^{[1]}$,$\boldsymbol{\lambda }^{[1]}$,$\boldsymbol{W}^{[1]}$, $\boldsymbol{Z}^{[1]}$,$\boldsymbol{\mu }^{[1]}$,$\boldsymbol{\sigma }^{[1]2}$,$\boldsymbol{\alpha }^{[1]}$,$\boldsymbol{\eta }^{[1]2}$, and compute $\boldsymbol{\phi }^1$.
2. Sample
  $$ \begin{align*} \gamma^{[t]} \sim Beta\left(\sum_{j=1}^n I\left(r^{[t-1]}_j=1\right)+1, \sum_{j=1}^n I\left(r^{[t-1]}_j=0\right)+1\right). \end{align*} $$
3. Sample
  $$ \begin{align*}\kern-3pt \beta^{[t]} \sim Beta\left(\sum_{k=1}^s \sum_{j=1}^m I\left(w^{[t-1]}_{kj}=1\right)+1, \sum_{k=1}^s \sum_{j=1}^m I\left(w^{[t-1]}_{kj}=0\right)+1\right). \end{align*} $$
4. Sample
  $$ \begin{align*} \delta^{[t]} \sim Beta\left(\sum_{i=1}^n \sum_{j=1}^m I\left(z^{[t-1]}_{ij}=r^{[t-1]}_j\right)+1,\right. \\\left. \sum_{i=1}^n \sum_{j=1}^m I\left(z^{[t-1]}_{ij}=1-r^{[t-1]}_j\right)+1\right). \end{align*} $$
5. $$ \begin{align*} P_0^R &\triangleq \delta^{[t] \sum_{i=1}^n I(z^{[t-1]}_{ij}=0)} (1-\delta^{[t]})^{\sum_{i=1}^n I(z^{[t-1]}_{ij}=1)} (1-\gamma^{[t]}), \\ P_1^R &\triangleq \delta^{[t] \sum_{i=1}^n I(z^{[t-1]}_{ij}=1)} (1-\delta^{[t]})^{\sum_{i=1}^n I(z^{[t-1]}_{ij}=0)} \gamma^{[t]}, \end{align*} $$
  Sample
  $$ \begin{align*} r^{[t]}_j \sim \ i.i.d. \ Bernoulli\left(\frac{P_1^R}{P_0^R + P_1^R}\right), \ for \ j = 1,2,\dots,m. \end{align*} $$
6. $$ \begin{align*} \theta_k = \sum_{i=1}^n I\left(\lambda^{[t-1]}_{ik} = 1\right), \ for \ k =1,2,\dots,s, \end{align*} $$
  Sample
  $$ \begin{align*} \boldsymbol{P}^{[t]} \sim Dirichlet(\theta_1,\theta_2,\dots,\theta_s). \end{align*} $$
7. $$ \begin{align*} & P_k^{\lambda} \\ & \triangleq \prod_{j=1}^m \prod_{c=0}^1 \left[\frac{1}{\sqrt{2 \pi \eta^{[t-1]2}_c}} exp\left \{ - \frac{(y_{ij}-\alpha^{[t-1]}_c)^2}{2 \eta^{[t-1]2}_c} \right\} \right]^{I(z^{[t-1]}_{ij} \oplus w^{[t-1]}_{kj} = c)} \\ & \cdot p^{[t]}_k, \ k =1,2,\dots,s, \end{align*} $$
  Sample
  $$ \begin{align*} & \boldsymbol{\lambda}_{i}^{[t]} =(\lambda_{i1},\dots,\lambda_{ik},\dots,\lambda_{is}) \\ & \sim Multinomial(1;\frac{P_1^{\lambda}}{\sum_{k=1}^s P_k^{\lambda}},\frac{P_2^{\lambda}}{\sum_{k=1}^s P_k^{\lambda}},\dots,\frac{P_s^{\lambda}}{\sum_{k=1}^s P_k^{\lambda}}), \\ & i=1,2,\dots,n. \end{align*} $$
8. $$ \begin{align*} & P_0^W \\ &\triangleq \prod_{i=1}^n \prod_{c=0}^1 \left[\frac{1}{\sqrt{2 \pi (\eta^{[t-1]}_c})^2} exp \left\{ - \frac{(y_{ij}-\alpha^{[t-1]}_c)^2}{2 (\eta^{[t-1]}_c)^2} \right\} \right] ^ {I\left(z^{[t-1]}_{ij} = c\right)} \\ & \cdot (1-\beta^{[t]}), \\ & P_1^W \\ &\triangleq \prod_{i=1}^n \prod_{c=0}^1 \left[\frac{1}{\sqrt{2 \pi (\eta^{[t-1]}_c)^2}} exp\left \{ - \frac{(y_{ij}-\alpha^{[t-1]}_c)^2}{2 (\eta^{[t-1]}_c)^2} \right\} \right]^{I\left(z^{[t-1]}_{ij} \oplus \lambda^{[t]}_{ik} = c\right)} \\ & \cdot \beta^{[t]}, \end{align*} $$
  Sample
  $$ \begin{align*} w_{kj}^{[t]} \sim \ i.i.d. \ Bernoulli\left(\frac{P_1^W}{P_0^W + P_1^W}\right), \\ for \ k = 1,2,\dots,s, \ j = 1,2,\dots,m. \end{align*} $$
9. $$ \begin{align*} & P_0^Z \\ &\triangleq \prod_{c=0}^1 \left[\frac{1}{\sqrt{2 \pi (\eta^{[t-1]}_c)^2}} exp \{ - \frac{(y_{ij}-\alpha^{[t-1]}_c)^2}{2 (\eta^{[t-1]}_c)^2} \} \right] ^{I\left(\sum_{k=1}^S \lambda^{[t]}_{ik} w_{kj} = c\right)} \\ & \cdot \left[\frac{1}{\sqrt{2 \pi (\sigma^{[t-1]}_0)^2}} exp\left \{ - \frac{(x_{ij}-\mu^{[t-1]}_0)^2}{2 (\sigma^{[t-1]}_0)^2} \right\} \right] \\ & \cdot \delta^{[t]I(r_j=0)} (1 - \delta^{[t]})^{I(r_j=1)}, \\ & P_1^Z \\ & \triangleq \prod_{c=0}^1 \left[\frac{1}{\sqrt{2 \pi (\eta^{[t-1]}_c)^2}} exp\left \{ - \frac{(y_{ij}-\alpha^{[t-1]}_c)^2}{2 (\eta^{[t-1]}_c)^2} \right\} \right] ^ {I\left(1 \oplus \sum_{k=1}^S \lambda^{[t]}_{ik} w_{kj} = c\right)} \\ & \cdot \left[\frac{1}{\sqrt{2 \pi (\sigma^{[t-1]}_1)^2}} exp\left \{ - \frac{(x_{ij}-\mu^{[t-1]}_1)^2}{2 (\sigma^{[t-1]}_1)^2} \right\} \right] \\ & \cdot \delta^{[t]I(r_j=1)} (1 - \delta^{[t]})^{I(r_j=0)}, \\ \end{align*} $$
  Sample
  $$ \begin{align*} z_{ij}^{[t]} \sim \ i.i.d. \ Bernoulli\left(\frac{P_1^Z}{P_0^Z + P_1^Z}\right), \\ for \ i = 1,2,\dots,n, \ j = 1,2,\dots,m. \end{align*} $$
10. Sample
  $$ \begin{align*} & \mu^{[t]}_c \sim N\left(\frac{\sum_{i=1}^n \sum_{j=1}^m (x_{ij} \cdot I(z^{[t]}_{ij}=c) )}{\sum_{i=1}^n \sum_{j=1}^m I(z^{[t]}_{ij}=c)},\frac{\sum_{i=1}^n \sum_{j=1}^m \sigma_c^{[t-1]2}} {\sum_{i=1}^n \sum_{j=1}^m I(z^{[t]}_{ij}=c)}\right) \\ & \cdot I(\mu^{[t]}_c \in [-50,50]), \ for \ c = 0, 1. \end{align*} $$
11. Sample
  $$ \begin{align*} \sigma_c^{2[t]} \sim & inv\Gamma\left(\frac{\sum_{i=1}^n \sum_{j=1}^m I(z^{[t]}_{ij}=c)}{2}-1\right., \\ & \left.\frac{\sum_{i=1}^n \sum_{j=1}^m ((x_{ij}-\mu^{[t]}_c)^2 \cdot I(z^{[t]}_{ij}=c) )}{2}) \cdot I(\sigma_c^{2[t]} \in [0,100]\right), \\ & for \ c = 0, 1. \end{align*} $$
12. Compute $\boldsymbol{\phi }^{[t]}= z^{[t]}_{ij} \oplus \sum _{k=1}^S \lambda ^{[t]}_{ik} w^{[t]}_{kj}.$
13. Sample
  $$ \begin{align*} \alpha^{[t]}_c & \sim N\left(\frac{\sum_{i=1}^n \sum_{j=1}^m (y_{ij} \cdot I(\phi^{[t]}_{ij}=c) )}{\sum_{i=1}^n \sum_{j=1}^m I(\phi^{[t]}_{ij}=c)},\right.\\ & \left.\frac{\sum_{i=1}^n \sum_{j=1}^m \eta_c^{[t-1]2}}{\sum_{i=1}^n \sum_{j=1}^m I(\phi^{[t]}_{ij}=c)}\right) \cdot I(\alpha^{[t]}_c \in [-50,50]), \\ & for \ c = 0, 1.\end{align*} $$
14. Sample
  $$ \begin{align*} \eta_c^{[t]2} \sim & inv\Gamma\left(\frac{\sum_{i=1}^n \sum_{j=1}^m I(\phi^{[t]}_{ij}=c)}{2}-1,\right. \\ & \left.\frac{\sum_{i=1}^n \sum_{j=1}^m ((x_{ij}-\alpha^{[t]}_c)^2 \cdot I(\phi^{[t]}_{ij}=c) )}{2}\right)\\ & \cdot I(\eta_c^{[t]2} \in [0,100]), \\ & for \ c = 0, 1. \end{align*} $$

### A.2 Sensitivity analysis

We conduct sensitivity analysis to investigate the influence of the truncated uniform priors. The truncation boundaries for $\mu _c$ and $\alpha _c$ is set to be (-50,50), (-100,100) and (-200,200), respectively, and that for $\sigma _c^2$ and $\eta _c^2$ is (0,100), (0,200) and (0,400). The experiment results illustrate the choices of truncation boundary have little effect on the performance of algorithm BaySub.

### A.3 Comparison on computational cost

For Simulation 1 to Simulation 3 in this paper, both the K-means, K-medoids and Hierarchical Clustering methods only take about 1 min, while BaySub takes about 10 iterations (about 90 s) to converge and 200 iterations (about 28 min) to collect posterior sample according to Figure 5, although it is time-consuming to perform Bayesian inference by MCMC for large datasets and high-dimensional models. Consider the datasets are offline and MCMC methods can provide the full picture of posterior distribution, it is acceptable.
